# Cytomegalovirus Impairs the Induction of Indoleamine 2,3-Dioxygenase Mediated Antimicrobial and Immunoregulatory Effects in Human Fibroblasts

**DOI:** 10.1371/journal.pone.0064442

**Published:** 2013-05-15

**Authors:** Kathrin Heseler, Silvia K. Schmidt, Katrin Spekker, Christian Sinzger, Rüdiger V. Sorg, Marc Quambusch, Albert Zimmermann, Roland Meisel, Walter Däubener

**Affiliations:** 1 Institute of Medical Microbiology and Hospital Hygiene, Heinrich-Heine-University, Düsseldorf, Germany; 2 Institute for Virology, Medical Center, University of Ulm, Ulm, Germany; 3 Institute for Transplantation Diagnostics and Cell Therapeutics, Heinrich-Heine-University, Düsseldorf, Germany; 4 Institute for Virology, Heinrich-Heine-University, Düsseldorf, Germany; 5 Clinic for Pediatric Oncology, Hematology and Clinical Immunology, Center for Child and Adolescent Health, Heinrich-Heine-University, Düsseldorf, Germany; Université Paris Descartes, France

## Abstract

Human fibroblasts provide immunosuppressive functions that are partly mediated by the tryptophan-catabolizing enzyme indoleamine-2,3-dioxygenase (IDO). Moreover, upon stimulation with inflammatory cytokines human fibroblasts exhibit broad-spectrum antimicrobial effector functions directed against various clinically relevant pathogens and these effects are also IDO-dependent. Therefore human fibroblasts are suggested to be involved in the control of immune reactions during infectious diseases. As human cytomegalovirus (HCMV) represents a pathogen frequently found in immunocompromised hosts and IDO is involved in the control of HCMV growth, we here investigated the impact of HCMV infection on IDO-mediated antimicrobial and immunoregulatory effects. We show that infection with HCMV substantially impairs IFN-γ-induced IDO-activity in human fibroblasts in a dose and time dependent fashion. Consequently, these cells are no longer able to restrict bacterial and parasitic growth and, furthermore, loose their IDO-mediated immunosuppressive capacity. Our results may have significant implications for the course of HCMV infection during solid organ transplantation: we suggest that loss of IDO-mediated antimicrobial and immunoregulatory functions during a HCMV infection might at least in part explain the enhanced risk of organ rejection and infections observed in patients with HCMV reactivation after solid organ transplantation.

## Introduction

Human Cytomegalovirus (HCMV) causes chronic infection in >60% of the human population. The first contact with the virus usually occurs within the first two decades of life. Immunocompetent individuals rarely develop a febrile, infectious-mononucleosis like illness with fever, leukopenia and hepatitis, while most individuals stay asymptomatic during primary infection. A symptomatic, life-threatening primary infection is only described in neonates with an immature immune system or in individuals with severe immune deficiencies. The benign clinical outcome of primary infection is due to a robust immune response directed against human cytomegalovirus. It was found that beside classical T cells [1] γ/δ T cells [2] and NK cells [3] are involved in this defence. In addition to cellular immune reactions antibodies also participate in the HCMV-directed host defense. For example a serum antibody response to glycoprotein B has been identified which is linked to HCMV neutralising effects [4].

However, all these potent immune reactions fail to eliminate HCMV from the human body during primary infection. The virus persists in cells of different organs for the lifetime of the host and the cellular sites of viral latency become reservoirs of reactivation. It has been described that CMV has developed a couple of different immune escape mechanisms to counteract immune responses thus favouring the development of viral latency. For example: CMV is able to interfere with the MHC-I pathway of antigen processing and therefore blocks the activation and function of CD8 positive cells [5] thus favouring virus replication. Furthermore, T-cells do not only mediate cytotoxic effects since T-cell derived cytokines e. g. IFN-γ are important in the control of virus growth. For example Bodaghi *et al.* [6] showed that IFN-γ induces an antiviral effect directed against HCMV in human retinal pigment epithelial cells. In addition we found that IFN-γ-mediated induction of indoleamine 2,3-dioxygenase (IDO) is an antiviral effector mechanism directed against HCMV in human mesenchymal stromal cells [7]. Thus antiviral effects induced by IFN-γ are important for an effective antiviral response directed against HCMV. To escape the IFN-γ induced antiviral effects HCMV has developed several strategies to inhibit IFN-γ signalling during co-evolution within its specific host [8], [9], [10]. Since IFN-γ signalling is necessary to induce IDO mediated peripheral tolerance and antimicrobial effects [11] we analysed the capacity of HCMV to inhibit IFN-γ-mediated IDO activation and subsequent antimicrobial and immunoregulatory effects. In our *in vitro* study we used human foreskin fibroblasts (HFF) since these cells exert profound antimicrobial [12] and immunoregulatory [13] effects and in contrast to most other cell types support an efficient HCMV growth *in vitro*. In co-infection models we used *Toxoplasma gondii* and *Staphylococcus aureus* since both pathogens, like HCMV, are of particular importance in immunosuppressed patients. We found that HCMV is a major regulator of IDO activity in human fibroblasts and drastically reduces the antimicrobial and immunosuppressive properties of HFF. We suggest that HCMV-mediated inhibition of IDO activity may underlie the increased risk of graft rejection observed in transplant patients with HCMV reactivation [14]. In addition, the enhanced infection rate, reported in patients with symptomatic HCMV disease [15], might be partly explained by IDO inhibition.

## Materials and Methods

### Primary Cells, Cell Lines and Reagents

Human foreskin fibroblasts (HFF) and OKT3 cells (Monoclonal-antibody-producing hybrid cell line producing antibodies against CD3, Ortho Pharmaceutical Corporation) cells were obtained from the American Type Culture Collection (Rockville, USA). Recombinant human IFN-γ was purchased from R&D Systems (Wiesbaden, Germany). L-tryptophan, L-kynurenine, 1-L-methyl-tryptophan (1-MT) and Ehrlich’s reagent were ordered from Sigma-Aldrich (Deisenhofen, Germany), DAPI was obtained from Boehringer (Ingelheim, Germany).

### Ethics Statement

This study obtained ethics approval from the ethics committee of the Medical Faculty of the Heinrich-Heine-University Düsseldorf (study no. 3838). Human peripheral blood mononuclear cells (PBMC) were generated from the blood of 5 healthy individuals (3 male and 2 female, average age 35 years) after informed and written consent.

### Human Cytomegalovirus

HCMV strains AD169 and TB40E were kindly provided by C. Sinzger (Institute for Medical Virology, Tübingen, Germany) and A. Zimmermann (Institute for Virology, Düsseldorf, Germany). Before infection of HFF, the virus-containing solution was thawed and diluted in tryptophan-free RPMI 1640 medium to reach a multiplicity of infection (MOI) of 0.3–10. In some experiments UV-inactivated HCMV preparations were used.

### IDO Assay

The enzyme activity of IDO directly correlates with the concentration of kynurenine in supernatants of tissue culture cells. Thus measurement of kynurenine can be used to determine IDO activity [16]. HFF (0.5–3×10^4^ per well) were plated in 96-well flat-bottomed microtiter plates in IMDM containing 5% FCS and 0.6 mM L-tryptophan. The cultures were stimulated with IFN-γ at concentrations from 1,000 U mL^−1^ to zero. The plates were incubated at 37°C and after 72 h kynurenine was detected in culture supernatants using Ehrlich’s reagent as described [16]. Data were given as mean kynurenine content of triplicate cultures. In some experiments IDO was induced by co-culturing HFF with OKT3 stimulated peripheral blood lymphocytes for three days. As a control 1-L-MT (1.5 mM) or a neutralizing anti- IFN-γ antibody (10 ng/mL) was added at the time point of HFF stimulation. In addition IDO protein and IDO mRNA were detected in stimulated cells using real-time PCR and western blot analysis as described [7].

### Toxoplasma Gondii

Tachyzoites of the BK strain obtained from Dres. Seitz and Saathoff, (Institute for Medical Parasitology, Bonn, Germany) were maintained in L929 murine fibroblasts (American Type Culture Collection, Rockville, USA). After IFN-γ stimulation for 72 hours and HCMV infection at different time points, HFF (3×10^4^ per well) were infected with 2×10^4^ toxoplasma tachyzoites per well. Toxoplasma growth was measured by the [^3^H]uracil incorporation method as described [7] and intracellular staining of parasites in HFF cells was performed as described [17].

### Staphylococcus Aureus

IFN-γ activated HFF were infected with 10–100 cfu/well. Bacterial growth was monitored after further incubation of 16 h by measuring optical density at 620 nm [7]. Bacteria were obtained from a routine diagnostic specimen. They were identified by colony morphology and positive coagulase reaction and in addition confirmed biochemically with a commercial system (API-20staph; bioMerieux, Lyon, France).

### T Cell Proliferation Assay

1**–**1.5×10^5^ peripheral blood mononuclear cells (PBMC), obtained from heparinised blood of healthy donors after Ficoll-Hypaque purification [18], were stimulated with a monoclonal anti-CD3 antibody (OKT3, American Type Culture Collection, Rockville, USA) in the presence of different amounts of fibroblasts as described [19]. In some experiments fibroblasts (0.5–2×10^4^ per well) were infected with HCMV and/or stimulated with IFN-γ at the start of the culture. After three days the cultures were pulsed with 0.2 µCi [^3^H]thymidine for 24 hours. T cell proliferation was measured by [^3^H]thymidine incorporation using liquid scintillation spectrometry (1205 Betaplate, PerkinElmer, Jugesheim, Germany). In some experiments IFN-γ content was determined in culture supernatants three days after OKT3 stimulation using Quantikine ELISA (R&D Systems, Wiesbaden, Germany) according to the instructions of the supplier.

### Statistical Analysis

All data are given as mean +/− SEM of 2–5 independent experiments and each experiment was performed in triplicates. Data of single experiments, also performed in triplicates, were given as mean +/− SD. For the comparison of different data the Students t-test for unpaired groups was used and the p-value was calculated using GraphPad Prism software.

## Results

### Inhibition of IFN-γ-mediated IDO Induction in Human Fibroblasts by HCMV

Human cytomegalovirus is well known to subvert antiviral immunity partly by interfering with IFN-γ-mediated host defense mechanisms. We thus explored the potential impact of HCMV infection on IFN-γ-induced IDO induction and resulting antimicrobial and immunoregulatory effects in human fibroblasts.

**Figure 1:**
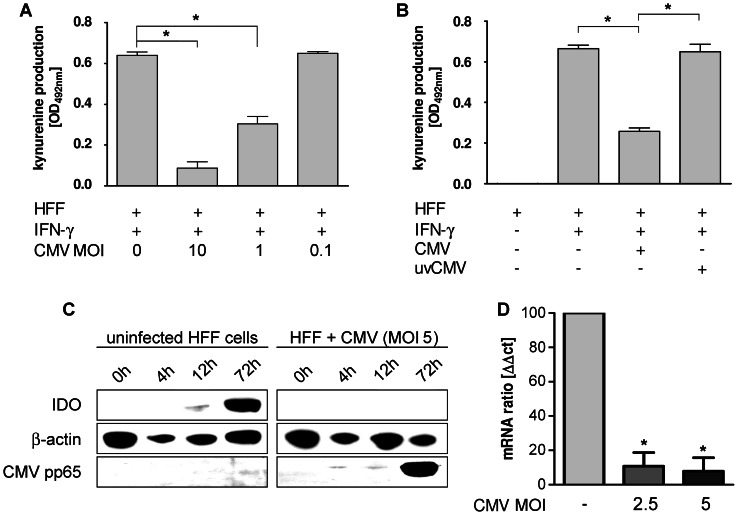
Inhibition of IDO-activity in HCMV infected human fibroblasts. HFF (3×10^4^/well) were stimulated with IFN-γ (600 U/ml) for three days. (A) Different amounts of HCMV (CMV) or (B) HCMV or UV-treated HCMV (uvCMV) corresponding to MOI 5 [multiplicity of infection] were added together with IFN-γ. Kynurenine concentration in the supernatant was determined to directly measure IDO enzyme activity using Ehrlich’s reagent three days later. Data are given as mean kynurenine production +/− SEM of three independent experiments, each done in triplicates. The optical density measured in the negative control with unstimulated HFF is subtracted in all groups. Infection with viable, but not UV-treated HCMV, results in a significant reduction (p<0.05) of IFN-γ-induced IDO activity. (C) HFF (1×10^6^) were stimulated with IFN-γ (600 U/ml) in the absence or presence of HCMV (MOI 5). Cells were harvested at different time points after viral infection and IDO protein was detected in western blot analysis. While β-actin was utilized as a protein loading control, the viral pp65 protein served as an infection control. (D) HFF (1×10^6^) were stimulated with IFN-γ in the presence or absence of HCMV (MOI 2.5 or 5). After 24 hours cells were harvested and IDO and β-actin mRNA was quantified using real-time PCR. Data are given as % of positive control (without HCMV) +/− SD of three independent experiments. A significant reduction of IDO mRNA is indicated by asterisks.

The stimulation with IFN-γ induced a profound IDO activity in human foreskin fibroblasts (HFF). IDO activity, however, was strongly reduced, when cells were infected with HCMV. The two different HCMV strains used (AD169 and TB40E) had a comparable capacity to inhibit IDO activity (data not shown). Furthermore, the magnitude of the IDO inhibitory effect was critically depended on the infectious dose (Figure 1A), and on the replication competence of the virus preparation since UV-inactivated virus preparations were unable to reduce IFN-γ-induced IDO activation (Figure 1B). Western blot analysis, depicted in Figure 1C, showed a strong reduction of IDO protein in IFN-γ-activated HFF when cells were infected with HCMV. Likewise the expression of IDO mRNA is substantially reduced in HCMV-infected human fibroblasts (Figure 1D). Taken together, these data provide compelling evidence that HCMV infection is able to substantially impair IDO protein biosynthesis and IDO enzyme activity in human fibroblasts.

**Figure 2:**
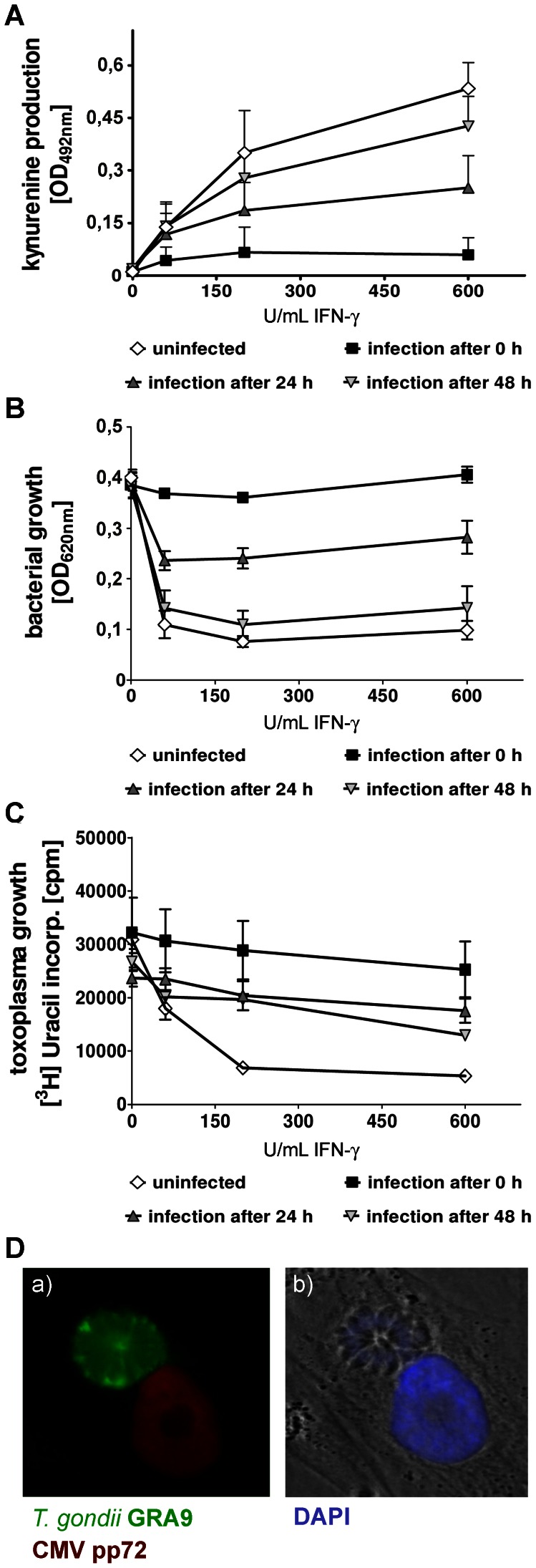
HCMV infection abrogates IFN-γ-induced IDO activity and subsequent antimicrobial effects. (A) HFF (3×10^4^/well) were stimulated with different amounts of IFN-γ in cell culture medium containing 0.6 mM tryptophan. The cultures were infected with HCMV (MOI 5) at the time point of IFN-γ stimulation, after 24 hours or 48 hour, respectively. After three days the kynurenine production by the cells was determined to directly measure IDO enzyme activity using Ehrlich’s reagent. Data are given as mean kynurenine production +/− SEM of five independent experiments, each done in triplicates. The OD measured in the negative control (unstimulated HFF) is subtracted in all groups. (B) HFF were stimulated with IFN-γ and infected with HCMV as described above. After three days cultures were infected with *S. aureus* (10–100 cfu/well) and bacterial growth was determined photometrically 24 hours later. Data are given as mean optical density +/− SEM of three independent experiments, each done in triplicates. (C) HFF were stimulated with IFN-γ and infected with HCMV as described above. Three days after activation the cells were infected with *T. gondii* (2×10^4^/well) and parasite growth was determined using [^3^H]-uracil three days later. Data are given as mean cpm +/− SEM of four independent experiments, each done in triplicates. HCMV added within the first 24 h of culture significantly reduced (p<0.05) IFN-γ-induced IDO activity and subsequent antibacterial and antiparasitic effects. (D) HFF cultures were co-infected with *T. gondii* and HCMV. After two days an immunofluorescence analysis was performed. While *T. gondii* parasites were detected with an anti-GRA9 antibody stained in green within the parasitophorous vacuole, HCMV was detected with an anti-HCMV-pp72 antibody stained in red [panel a)] within the nucleus of the host cell. As a control panel b) shows DAPI nuclear staining in blue with merged phase contrast.

Additional experiments, shown in Figure 2A indicate that the magnitude of this inhibitory effect is also dependent on the time point of infection: the addition of HCMV together with IFN-γ had the strongest impact, while HCMV infection 48 h after IFN-γ stimulation exerted only moderate inhibitory effects.

### Inhibition of IDO-mediated Antimicrobial Effects in Human Fibroblasts by HCMV

IDO is a potent inducer of antimicrobial effects in several human cell types. To investigate the influence of HCMV on IDO-mediated antibacterial effects, we stimulated HFF with IFN-γ for 72 h and added HCMV at different time points. As shown in Figure 2B IFN-γ-activated HFF cells were able to inhibit the growth of *Staphylococcus aureus*. An infection of fibroblasts with HCMV, however, results in a dramatic inhibition of this antibacterial effect. Again, the time point of HCMV infection proved critical for the magnitude of antibacterial effects. The observed “protective” effect of HCMV on bacterial growth followed a similar kinetic as the HCMV-mediated inhibition of IDO activity depicted in Figure 2A. Comparable data were obtained in infection experiments using *Toxoplasma gondii*, which is an obligate intracellular parasite and only replicates in viable cells. As shown in Figure 2C *T. gondii* is well able to replicate in HFF which are heavily infected with HCMV, thus excluding the possibility that the observed HCMV-mediated inhibitory effects on IDO and resulting protective effects on microbial growth are simply due to viral killing of fibroblasts. Furthermore, immunofluorescence analysis shown in Figure 2D demonstrates that HCMV and *T. gondii* are capable of replicating in one and the same fibroblast at the same time. CMV (pp72, red staining) replicates in the nucleus of the cell, whereas the parasitophoric vacuole containing *T. gondii* parasites (GRA9, green staining) is found in the perinuclear region. The associated phase contrast picture shows the blue DAPI stain of the parasite nuclei within the parasitophorous vacuole and additionally the nucleus of the cell.

### Influence of HCMV on IDO Mediated Effects in Co-cultures of HFF and Activated T Cells

**Figure 3:**
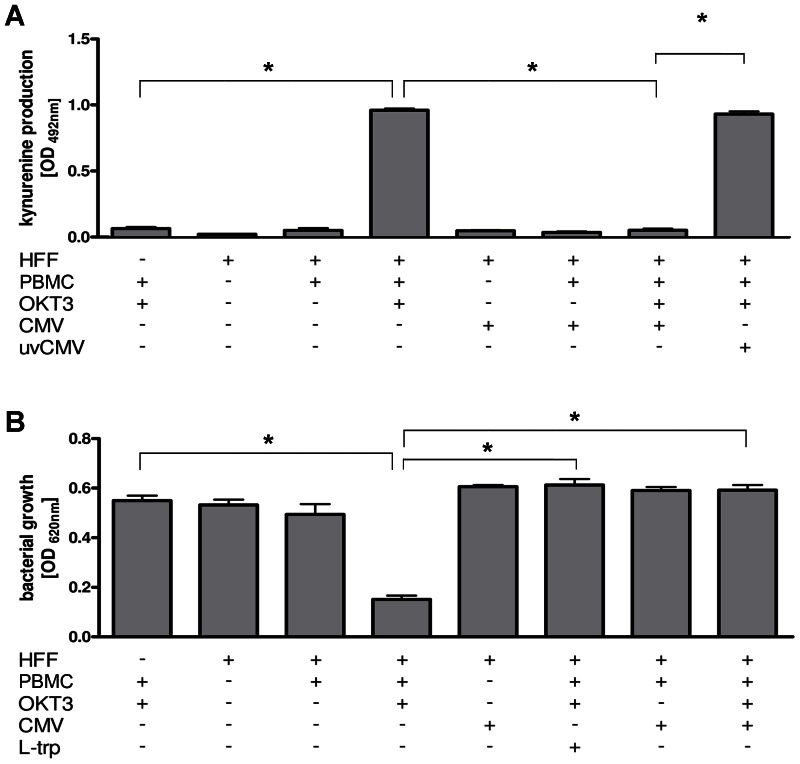
HCMV blocks IDO activity and subsequent antibacterial effects observed in co-cultures of activated T cells and HFF. Peripheral blood mononuclear cells (PBMC; 1×10^5^/well), stimulated with a CD3-directed mAb (OKT3), were co-cultured with HFF (2×10^4^/well) in the absence or presence of HCMV (MOI 5). As control UV-inactivated HCMV (uvCMV) was used. (A) After three days IDO activity was determined and is presented as mean kynurenine production +/− SEM of 4 independent experiments, each done in triplicates. The OD measured in the negative control (unstimulated HFF) is subtracted in all groups. (B) HFF/PBMC co-cultures were set up as described above. After three days cultures were infected with *S. aureus* (10–100 cfu/ml) and bacterial growth was determined photometrically 24 hours later. As a control L-tryptophan (100 µg/ml) was added at the time point of bacterial infection. Data are given as mean OD_(620 nm)_ +/− SEM of three experiments, each done in triplicates. Significant differences (p<0.05) as compared to the positive control are marked by asterisks.

We found that IDO induction in HFF by recombinant IFN-γ, as well as subsequent antimicrobial effects were inhibited in the presence of HCMV. In order to mimic the *in vivo* situation more closely we cultured HFF, either HCMV infected or not, together with peripheral blood mononuclear cells (PBMC) stimulated with antibodies directed against CD3. As shown in Figure 3A we found a strong IDO induction when HFF were cultured in the presence of CD3-activated PBMC which was significantly blocked when HCMV was present in the culture system. However, supernatants of unstimulated cultures or of PBMC cultures activated in the absence of HFF did not contain detectable amounts of kynurenine. As expected, UV-inactivated HCMV preparations had no impact on PBMC-induced IDO activity in HFF. Thus, only infection with replication-competent HCMV substantially impairs IFN-γ-induced IDO expression in human fibroblasts (Fig. 3A). In addition, we found that HFF cultured in the presence of OKT3 stimulated PBMC were able to restrict the growth of *Staphylococcus aureus* and this antibacterial effect could be blocked by the addition of excess amounts of tryptophan or by co-infection with HCMV (Fig. 3B). In order to exclude a possible direct inhibition of IFN-γ production by T-cells stimulated in the presence of HCMV, we determined the amount of IFN-γ in culture supernatants of OKT3 stimulated PBMC, that were cultured in the presence or absence of HFF and or HCMV (MOI 5). In all culture supernatants harvested three days after OKT3 stimulation we found equal amounts of IFN-γ (about 37 ng/ml).

**Figure 4:**
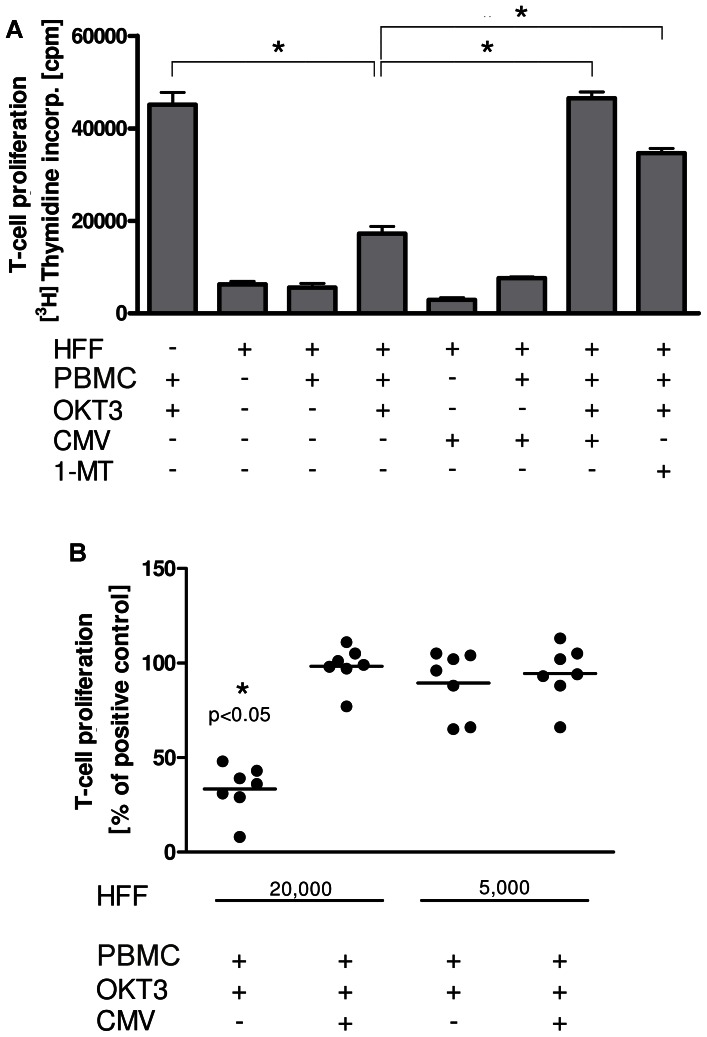
HCMV infection abrogates the immunosuppressive effect of human fibroblasts . (A) PBMC (2×10^5^/well) were activated with a CD3-directed mAb (OKT3) and cultured in the presence or absence of HFF which were infected with HCMV (MOI 5) or not. After three days T cell proliferation was assessed using [^3^H]-thymidin. Data are given as mean cpm +/− SD of a representative experiment performed in triplicates. As a control 1- MT was used. (B) Different amounts of HFF, either HCMV infected (MOI 5) or not, were co-cultured with OKT3-activated PBMC. Thereafter T cell proliferation was determined as described above. Data are given as % of positive control without HFF. Each dot represents a single experiment (n = 7), each performed in triplicates. A significant inhibition of T cell proliferation by HFF is marked with asterisks.

Human fibroblasts are able to restrict the proliferation of T cells in an IDO-dependent manner. Therefore, we set out to analyze the impact of HCMV infection of HFF on their immunosuppressive capacity. As shown in Figure 4A we found that the inhibitory effect of HFF on T-cell proliferation was significantly reversed in the presence of HCMV infection. To analyse the specific role of IDO in this effect we added the IDO inhibitor 1-MT and found that this substance was able to antagonise the immunosuppressive effect of HFF on T-cell proliferation, indicating that IDO is critically involved in this process.

In additional experiments we observed that the inhibitory effect of HFF on T-cell proliferation depends on the amount of HFF present in the co-culture system (Figure 4B). While 2×10^4^ HFF provided a strong inhibitory effect, 5×10^3^ HFF were unable to suppress T-cell proliferation. The addition of HCMV to cultures with high numbers of HFF antagonizes their T-cell inhibitory effect, while the same amount of HCMV added to low numbers of HFF did not alter T-cell proliferation thus ruling out an unspecific T cell stimulatory effect of HCMV. Together these data indicate that HCMV infection of human fibroblasts substantially impedes their antimicrobial and T cell inhibitory effector function.

## Discussion

In our present work we describe for the first time that HCMV is able to inhibit IFN-γ-induced, IDO-dependent antimicrobial and immunoregulatory effects in human fibroblasts. We demonstrate that this interaction between HCMV and IDO-mediated effects critically depends on intact virus, the time point of HCMV infection as well as the number of host cells and virus employed. HCMV is able to infect different cell types *in vivo*, however *in vitro* only a limited number of cell types including fibroblasts have been usually used to analyse HCMV growth. The discrepancy between cell types infected by HCMV *in vivo* and *in vitro* is at least in part due to selection processes during virus isolation. Therefore, most available viral strains were adapted to replicate in fibroblasts and fibroblast-like cells [20]. We show that HCMV is able to inhibit IDO expression in human fibroblasts stimulated with recombinant IFN-γ or by natural IFN-γ produced by activated T-cells. This inhibition is not a consequence of direct viral killing of fibroblasts as HCMV-infected cells do still support the replication of *T. gondii*, a process that requires viable host cells (Figure 2C, 2D). Rather, the HCMV-induced inhibitory effect is suggested to be due to a direct interference of HCMV with components of the intracellular IFN-γ signalling cascade. An interaction of HCMV with IFN-γ signalling was first described by Miller *et al.* [8]. This interference was found to be due to a reduced CIITA expression, an enhanced degradation of Jak1 or an insufficient phosphorylation of STAT1 and STAT2 [8], [9], [10].

In line with these published data on the interference of HCMV with IFN-γ-mediated signalling events we found that HCMV infection results in reduced IDO mRNA and protein expression as well as enzyme activity in human fibroblasts after IFN-γ stimulation. A comparable HCMV/IDO interaction was also found by Lopez *et al.* in placental tissue explants [21].

IFN-γ-induced IDO activity can exert substantial antimicrobial effector function. In human cells IDO mediates antibacterial effects against Chlamydia, group B streptococci, enterococci and *Staphylococcus aureus* [22]; antiviral effects against HCMV, HSV-1, HSV-2, vaccinia virus, measles virus; and antiparasitic effects against *Toxoplasma gondi* [12] and *Neospora caninum*. In this manuscript we analysed the influence of HCMV on the antimicrobial defense against *S. aureus* and *T. gondii*. All three pathogens are capable of inducing life-threatening infections in immunocompromised individuals and clinically apparent HCMV or *T. gondii* reactivation is also typically confined to this patient population. The clinical manifestation of *T. gondii* or HCMV reactivation is frequently observed in so called immunoprivileged organs, e.g. the eye and the brain, and both microorganisms can cause a life-threatening pneumonia. We thus speculate that the inhibition of IFN-γ-induced IDO activity by HCMV may result in an increased risk of developing secondary infections with *T. gondii* and other IDO-sensitive pathogens listed above. This might be of clinical relevance since observations in transplant patients indicate an enhanced risk of infections in patients with HCMV reactivation [15], [23], [24]. The fact that fibroblasts represent a cell type abundantly present in various tissues and organs throughout the human body supports the hypothesis that HCMV-mediated IDO inhibition might indeed impair antimicrobial host defense. However, based on the species-specificity of HCMV infection as well as IDO expression [7], murine models will unfortunately not be informative with regard to the *in vivo* impact of HCMV infection on IDO-mediated antimicrobial effects.

IDO, in addition to its role in antimicrobial defense, is also involved in the regulation of T-cell responses. Many data indicate that professional antigen-presenting cells such as dendritic cells are capable of mediating IDO-dependent immunoregulatory effects via tryptophan depletion, production of toxic kynurenines, induction of T-cell anergy and/or apoptosis or by induction of regulatory T cells [25]–[28]. However, non-professional phagocytes such as endothelial or epithelial cells, fibroblasts [13] and mesenchymal stromal cells [29] are also able to regulate T-cell activation in an IDO-dependent manner. Additionally, it was recently described that kynurenine, the downstream product of tryptophan, is a physiological ligand of the aryl hydrocarbon receptor (AhR). This finding is of special importance since the AhR is expressed on many different cell types and involved in the control of cell growth [28]. We have previously shown that the inhibition of T-cell growth in co-cultures could be blocked by an antibody against IFN-γ. Here we show in addition that the IDO-inhibitor 1-MT, which blocks several IDO mediated effects [30], is able to inhibit the HFF-mediated suppression of T-cell growth. This immunoregulatory effect is therefore at least in part mediated by IDO, but other immunoregulatory effector mechanisms described, for example the production of prostaglandins or of immunosuppressive cytokines, like IL-10 or TGF-β might also be involved. In accordance with our data on interference of HCMV infection with IFN-γ-induced antimicrobial effects in HFF, we observed substantial impairment of HFF-mediated immunosuppressive effects by HCMV infection, and this effect was critically dependent on the number of fibroblasts present in the co-culture system. Several *in vitro* an *in vivo* analysis have indicated that IDO is involved in the induction of tolerance and can mediate immunosuppression during organ transplantation [31], [32], [33]. A reactivation of HCMV after solid organ transplantation is a hazardous complication and frequently results in organ failure and rejection [34]. Recently Sadegi *et al.* [35] found signs of IDO activation in sera from kidney transplant recipients with activated cytomegalovirus infection. Based on our data we hypothesize that during HCMV reactivation after organ transplantation IDO-mediated immunosuppressive effects are at least in part blocked. This could result in an enhanced T-cell response which might be the cause of organ failure and rejection. Thus, strategies that aim at restoring full IDO expression in HCMV-infected fibroblasts may prove beneficial in this clinical scenario.

Given the fact that HCMV can act as an IDO inhibitor and might be able to enhance the risk of organ rejection it must be considered that the clinical use of IDO inhibitors (e.g. 1-methyl-tryptophan) could also result in an enhanced host versus graft reaction.
